# Mosaicism of the UDP-Galactose transporter *SLC35A2* in a female causing a congenital disorder of glycosylation: a case report

**DOI:** 10.1186/s12881-018-0617-6

**Published:** 2018-06-15

**Authors:** Kristen Westenfield, Kyriakie Sarafoglou, Laura C. Speltz, Elizabeth I. Pierpont, Joan Steyermark, David Nascene, Matthew Bower, Mary Ella Pierpont

**Affiliations:** 1 Department of Pediatrics, University of Minnesota, 2450 Riverside Avenue, Minneapolis, MN 55454 USA; 2 Divisions of Endocrinology, Genetics & Metabolism, University of Minnesota, 2450 Riverside Avenue, Minneapolis, MN 55454 USA; 3 Department of Neurology, Gillette Children’s Hospital, 200 University Avenue East, St. Paul, MN 55101 USA; 4 Division of Clinical Behavioral Neuroscience, Department of Pediatrics, University of Minnesota, 420 Delaware Street SE, Minneapolis, MN 55455-0392 USA; 5 University of Minnesota Masonic Children’s Hospital, 2450 Riverside Avenue, Minneapolis, MN 55454 USA; 6 Department of Radiology, University of Minnesota, 420 Delaware St. SE, Minneapolis, MN 55455 USA; 7 Molecular Diagnostics Laboratory, University of Minnesota Medical Center, 420 Delaware St SE, Minneapolis, MN 55455 USA; 8 Division of Genetics & Metabolism, Department of Pediatrics and Ophthalmology, 2450 Riverside Avenue, Minneapolis, MN 55454 USA

**Keywords:** Congenital disorder of glycosylation, Growth failure, Transferrin isoforms, *SLC35A2* mutation, Whole exome sequencing

## Abstract

**Background:**

Congenital disorders of glycosylation are rare conditions caused by genetic defects in glycan synthesis, processing or transport. Most congenital disorders of glycosylation involve defects in the formation or transfer of the lipid-linked oligosaccharide precursor of N-linked glycans. SLC35A2-CDG (previously CDG-IIm) is caused by hemizygous or heterozygous mutations in the X-linked gene *SLC35A2* that encodes a UDP-galactose transporter. To date there have only been 10 reported patients with *SLC35A2* mutations. Importantly, the patient presented here was not identified in infancy by transferrin isoform analysis, the most common testing to identify patients with a congenital disorder of glycosylation.

**Case presentation:**

A 27 month old girl with developmental delay, central hypotonia, cerebral atrophy, and failure to thrive with growth retardation was identified by whole exome sequencing to have a mosaic missense variant in *SLC35A2* (c.991G > A). This particular variant has been previously reported in a male as a mutation. Comparison of all clinical findings and new information on growth pattern, growth hormone testing and neurodevelopmental evaluation are detailed on the patient presented.

**Conclusion:**

This patient report increases the clinical and scientific knowledge of SLC35A2-CDG, a rare condition. New information on reduced growth, growth hormone sufficiency, lack of seizures, and neurodevelopmental status are presented. This new information will be helpful to clinicians caring for individuals with SLC35A2-CDG. This report also alerts clinicians that transferrin isoform measurements do not identify all patients with congenital disorders of glycosylation.

**Electronic supplementary material:**

The online version of this article (10.1186/s12881-018-0617-6) contains supplementary material, which is available to authorized users.

## Background

Congenital disorders of glycosylation (CDGs) are rare hereditary disorders associated with facial dysmorphism and neurological impairments including hypotonia, seizures, intellectual disability, and demyelinating neuropathy [1, 2]. There are two groups: one (CDG-I) that results from defects in glycan addition to the N-terminal; and a second group (CDG-II) that occurs due to defects in the processing of protein-bound glycans or vesicular transport [2, 3]. The most frequent of these is PMM2-CDG which includes 80% of the diagnosed cases [1].

SLC35A2-CDG (previously CDG-IIm) is a rare form of CDG caused by mutations in the X-linked gene *SLC35A2* that encodes a UDP-galactose transporter [4–6]. In eight patients with SLC35A2-CDG in the literature (Table 1), manifestations include seizures, failure to thrive, delayed myelination and cerebral atrophy [4, 6–8]. There are two other reports of *SLC35A2* variants in the literature, but no clinical information was provided for these cases, so they will not be discussed further [9, 10]. In this report we describe a female child with growth retardation, hypotonia and global developmental delay who was found by whole exome sequencing (WES) to be mosaic for an *SLC35A2* mutation previously reported [4].

**Table 1:** Clinical and Genetic Characteristics of 9 Children with *SLC35A2* Mutations

	Patient 1 (Current)	Patient 2 [4]	Patient 3 [4]	Patient 4 [4]	Patient 5 [7]	Patient 6 [6]	Patient 7 [6]	Patient 8 [6]	Patient 9 [8]
SLC35A2 cDNA	Mosaic	Mosaic	c.3G > A	Mosaic	c.797G > T	c.433_434del	c.972del	c.638C > T	c.950delG
	c.991G > A	c.15_91 + 48 delinsA		c.991G > A					
SLC35A2 Protein	p.Val331lle	p.Gly8Serfs*9	p.Met1?	p.Val331lle	p.Gly266Val	pTyr145Profs*76	p.Phe324Leufs*25	p.Ser213Phe	p.Gly317Alafs*32
Skewed X-inactivation	+	ND	ND	ND	+	+	+	ND	ND
Age at diagnosis (yr)	2	3	3	6	5	8	12	10	1
Sex	F	M	F	M	F	F	F	F	F
Ethnicity	Mexican/European	European	European	European	European	Asian	Asian	Asian	Asian
Microcephaly	–	–	+	+	–	ND	ND	ND	–
Brain MRI abnormality	Cerebral atrophy/delayed myelination	Small cerebellum	Delayed myelination/thin corpus callosum	Cerebral hypoplasia/atrophy	Cerebral atrophy/thin corpus callosum	Cerebral atrophy/delayed myelination	Enlarged lateral ventricle	Cortical & cerebral atrophy/thin corpus callosum	Cerebral & cerebellar atrophy/thin corpus callosum/enlarged lateral ventricle
Seizures	–	+	+	–	+	+	+	+	+
Hypsarrhythmia	–	+	+	–	+	+	+	+	+
Hypotonia	+	+	+	+	+	+	ND	+	+
Developmental delay	+	+	+	+	+	+	+	+	+
Facial dysmorphism	+	–	+	+	+	+	+	+	+
Ophthalmologic	Esotropia/astigmatism	Nystagmus	Retinitis pigmentosa	Ocular flutter	Blindness	ND	Eyeground white spot	ND	Heterochromia of iris/internal strabismus of right eye
Skeletal	–	–	Shortened extremities	Shortened extremities	Hypermobility/scoliosis	ND	ND	Hip dislocation	Small thumbs/tapering fingers/overlapping toes
Cardiac	–	ND	ND	ND	Small VSD/pericardial effusion/ASH	ND	ASD	ND	ND
Hematologic	–	+	–	–	–	–	–	–	–
GI	G-tube feeding	–	G-tube feeding	GERD/duodenal perforation	Pureed food only	ND	ND	ND	–
RecurrentInfections	–	–	+	–	+	ND	ND	ND	–
Renal	–	–	–	Acute nephrotic syndrome/dialysis	–	ND	ND	ND	–
Hypertension	–	–	–	+	ND	ND	ND	ND	ND
Hearing Reduced	–	ND	ND	ND	+	ND	ND	ND	ND

*ASD* atrial septal defect, *ASH* asymmetric septal hypertrophy, *GERD* gastroesophageal reflux disease, *ND* not described, *VSD* ventricular septal defect

## Case presentation

A female infant was delivered vaginally after a pregnancy complicated by hyperemesis and preterm labor. Birth weight was 2.89 kg (13th centile) and length was 53 cm (87th centile). At birth, resuscitation was required, followed by neonatal jaundice and feeding difficulties.

**Fig. 1:**
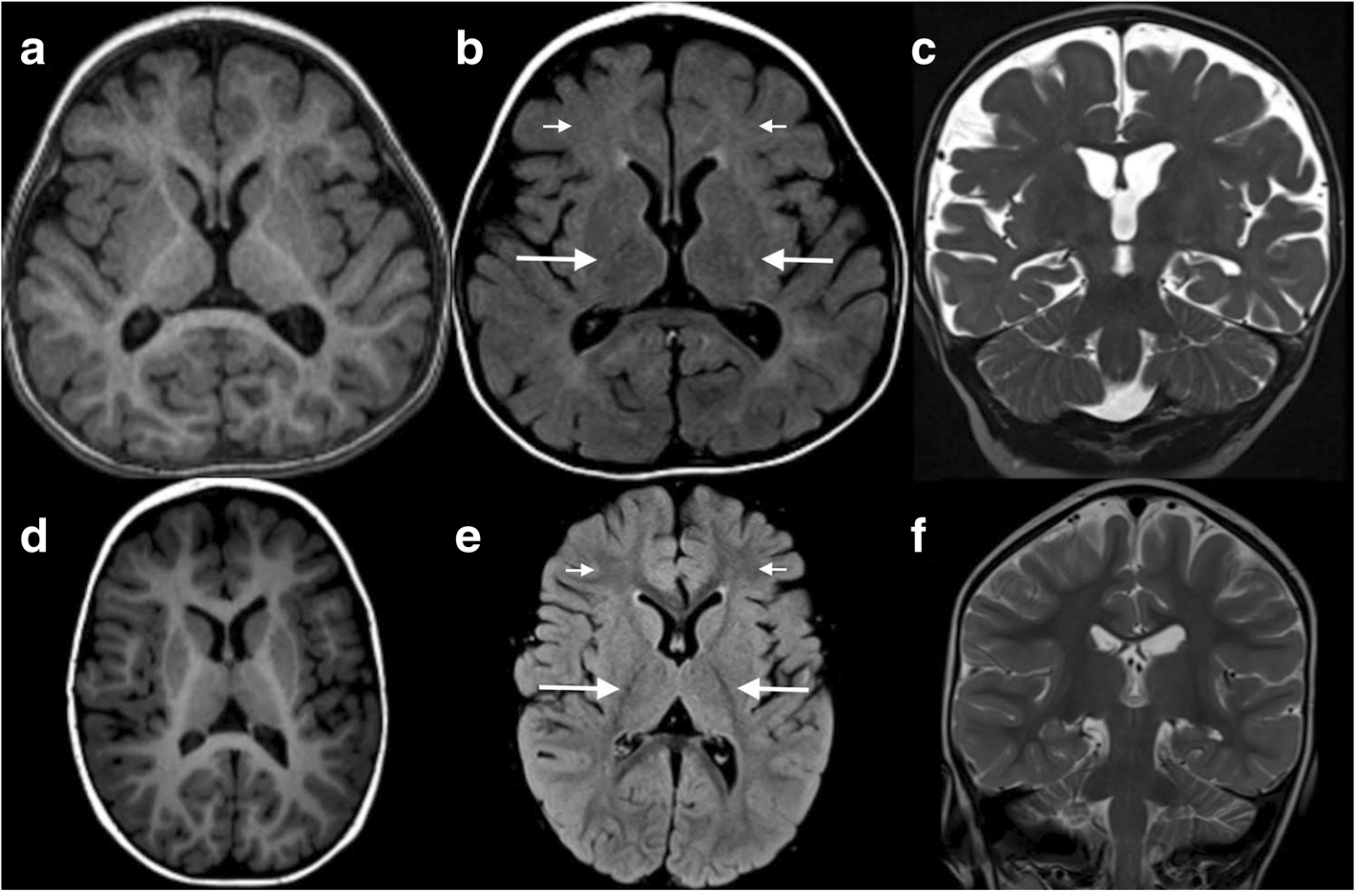
Brain MRI. **a**-**c**: SLC35A2-CDG patient at 22 months. **d**-**f**: Normal age and sex matched patient for comparison. **a**, **d** T1-weighted axial images, **b**, **e** TurboFLAIR axial images, **c**, **f** T2-weighted coronal images. Images a and b demonstrate mild widening of the cerebral sulci that is most notable in the frontal and temporal lobes. Shortening of the head in the anterior-posterior dimension (brachycephaly) was noted, which may be incidental as there are no other findings to suggest craniosynostosis. Image **c** also demonstrates cerebral volume loss. Delayed myelination is also best seen on **c**, exhibiting the lack of T2 hypointensity diffusely in the cerebral white matter (compared to **f**) expected for age. The delayed myelination is also evident on the TurboFLAIR image **b** as the lack of characteristic hypointensity in the posterior limbs of the internal capsules (long arrows) and in the deep white matter of the frontal lobes (short arrows)

During the first year of life, the patient had central hypotonia, gross motor delay, and failure to thrive. At age 9 months, brain magnetic resonance imaging (MRI) showed delayed myelination pattern on both T1- and T2-weighted images, generalized cerebral atrophy (white matter > grey matter), and mildly prominent extra-axial cerebrospinal fluid spaces consistent with a low brain volume (Fig. 1a-c). An electroencephalogram (EEG) obtained for staring spells was negative for clinical or subclinical seizures. Metabolic testing included normal lactate, pyruvate, free carnitine, CK, acylcarnitine profile, folate, vitamin B12, plasma amino acids, and very long chain fatty acids. Chromosomal microarray was normal. Methylation studies for Prader-Willi syndrome were normal. Transferrin isoforms by immunoaffinity liquid chromatography and electrospray mass spectrometry [11] at 12 months revealed elevated A-oligo/Di-oligo ratio of 0.02 (< 0.011) and Trisialo/Di-oligo ratio of 0.07 (< 0.05). Mono-oligo/Di-oligo, ApoCIII-1/ApoCIII-2, and ApoCIII-0/ApoCIII-2 ratios were within normal range. Repeat transferrin isoforms testing at 13.5 months was normal (Table 2). Due to weight loss and dehydration, a gastrostomy tube was placed at age 2 years. An ophthalmological examination showed esotropia, amblyopia, and hyperopic astigmatism. Otoacoustic emission testing was normal.

**Table 2:** Serum Transferrin in SLC35A2-CDG

	Method of Analysis of Transferrins	Result (Age < 12mo)	Later Result
Patient 1	Mass spectrometry	Slight increase of A-oligo/Di-oligo and Tri-sialo/Di-oligo ratios	Normal at 13.5 mo.
Patient 2 [4]	Mass spectrometry	Loss of galactose and sialic acid from multiple branches of complex N-glycans	Normal at 38 mo.
Patient 3 [4]	Mass spectrometry	Loss of galactose and sialic acid from multiple branches of complex N-glycans	Nearly normal at 36 mo.
Patient 4 [4]	Mass spectrometry	Loss of galactose and sialic acid from multiple branches of complex N-glycans	Normal at 5 yrs.
Patient 5 [7]	Isoelectric focusing	Abnormal type II CDG pattern with increased amounts of asialo, monosialo, disialo and trisialo compounds	Trend towards normal at 1–2 yrs. and abnormal at 5.2 yrs.
	Mass spectrometry		
Patient 6 [6]	Isoelectric focusing	ND	Normal at 8–10 yrs.
	Mass spectrometry		
Patient 7 [6]	Isoelectric focusing	ND	Normal at 8–10 yrs.
	Mass spectrometry		
Patient 8 [6]	Isoelectric focusing	ND	Normal at 8–10 yrs.
	Mass spectrometry		
Patient 9 [8]	Mass spectrometry	Normal at 12 mo.	Normal at 30 mo.

**Fig. 2:**
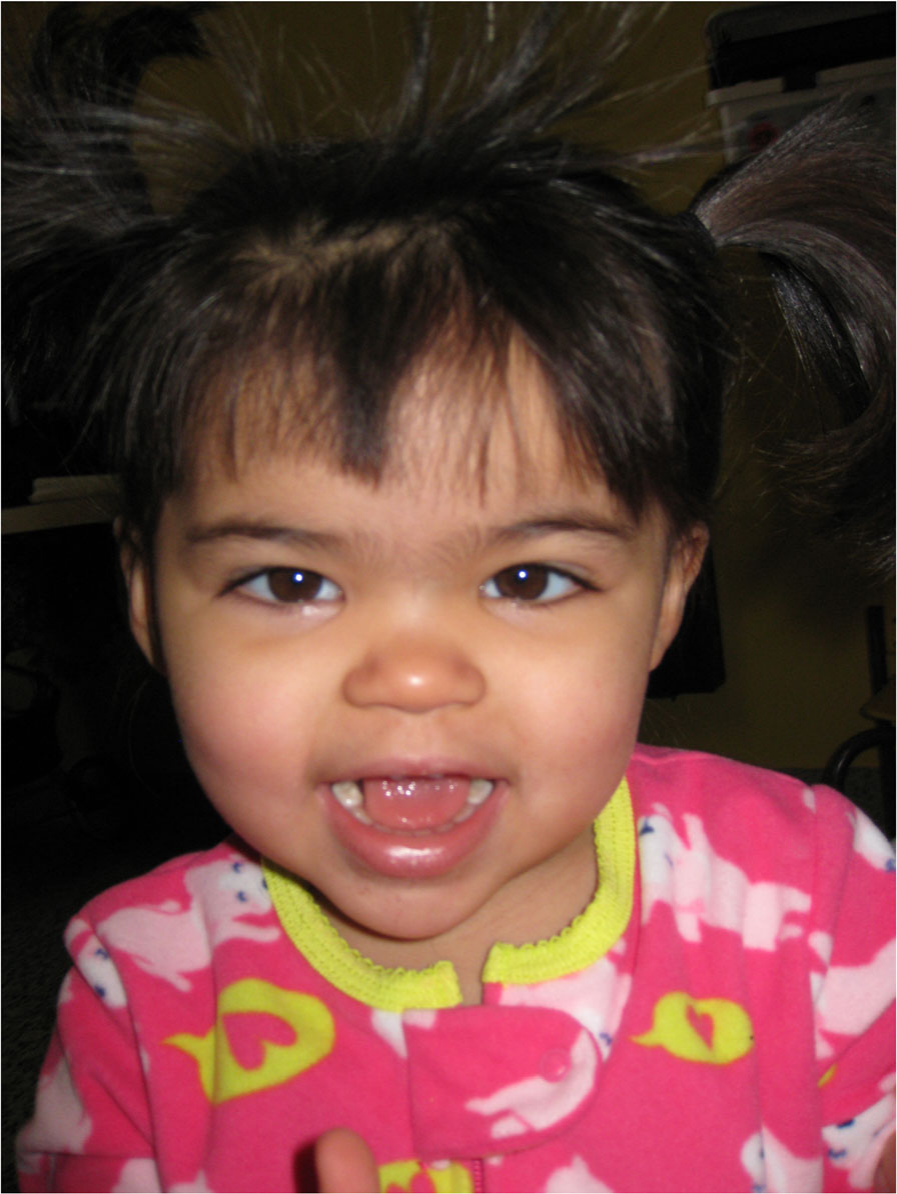
Facial appearance of patient 1 with *SLC35A2* mutation

At age 27 months, she was first seen at our institution. Examination revealed a weight of 10.4 kg (3rd centile), length of 78.4 cm (3rd centile) and OFC of 47.8 cm (50th centile). She had a high forehead, thick eyebrows with synophrys, midface hypoplasia, broad philtrum, thick lower lip, short palpebral fissures with deep-set eyes, bilateral epicanthal folds, flat nasal bridge, and a broad tip to the nose (Fig. 2). Other physical findings included prominent calcaneal fat pads anteromedial to the heels and a 2 cm irregular cafe au lait spot of the upper right groin area. A three-generation family history revealed that the patient’s mother was adopted, but the maternal family was of Mexican/European descent. The father’s family was said to be of European descent, but little else was known. There was no concern for consanguinity.

For WES, DNA was extracted from peripheral blood. The Agilent Clinical Research Exome Kit (Agilent, Santa Clara, CA) was used for sequence enrichment and the resulting libraries were sequenced on an Illumina HiSeq 2000 instrument (Illumina, San Diego, CA). Paired 100 base reads were mapped and genotyped using a custom informatics pipeline. The patient was found to have a mosaic variant c.991G > A or p.Val331lle (V331I) in the *SLC35A2* gene in 20% of reads (Additional file 1: Figure S1). The mother was negative for the c.991G > A variant in blood. This variant has been previously been reported as a mutation in the mosaic state in a male [4]. X-chromosome inactivation pattern was detected by methylation analysis of the androgen receptor (AR) gene locus by methylation-sensitive restriction digest, PCR and fragment analysis [12]. This revealed non-random skewed inactivation (ratio 85:15), where a ratio greater than 80:20 is considered as skewed inactivation.

Additional imaging studies were performed at 30 months of age including a renal ultrasound, ECG, and echocardiogram, all of which were normal. Other studies included an antithrombin III of 116% (85–135), thrombin time of 16.3 s (13–19), PTT of 34 (22–37 s) and INR of 1.03 (0.86–1.14), which were normal, and protein C of 118% (40–92), which was elevated. Because of her slow growth, IgA and IgG tissue transglutaminase antibodies were tested and both were normal. IgA level was 91 mg/dl (20–160). The liver transaminases and albumin (3.8 g/d (3.4–5) were normal except for mild elevated level of aspartate transaminase (AST) at 65 U/L (0–50).

At 34 months she underwent endocrine evaluation for growth failure. Her length was 81.8 cm (− 3.1 SDS), weight 10.6 kg (− 2.3 SDS) and head circumference was 49.3 cm (+ 0.59 SDS) (Additional file 2: Figure S2 A, B). Her growth hormone provocative stimulation test showed a peak growth hormone (GH) of 21 ng/ml (normal response > 10 ng/ml); insulin-like growth factor 1 (IGF-1) level was 65 ng/ml (16–178, − 0.1 SDS), insulin growth factor binding protein 3 (IGFBP3) was 3.1 μg/ml (0.8–3.9), and acid labile subunit (ALS) was slightly elevated at 11 mg/ml (1.9–10). Free T4 was 1.22 ng/ml (0.76–1.46) and TSH was 1.48 (0.4–4) mU/L.

In terms of neurocognitive development, the patient was delayed in reaching early milestones. She sat alone at age 15 months, crawled at 18 months, and walked independently at 2.5 years. She began speaking words after 2 years of age and started using word combinations at 3.5 years. A neurodevelopmental evaluation at age 53 months was conducted using the Bayley Scales of Infant and Toddler Development, Third Edition. Results indicated impaired performance on cognitive and language testing (19-to 22-month level). Expressive communication consisted primarily of single words and gestures, with some 2-and 3-word phrases. Fine motor function was at a 27-month level and gross motor function was at a 19-month level. The patient was socially engaged, cooperative, and good-natured during testing. Hyperactivity and distractibility were notable. Caregiver ratings of adaptive function were in the range of mild-to-moderate impairment. Although developmental progress was significantly slower than typical, the patient’s history indicated a pattern of consistent incremental gains over time, with beneficial response to early intervention services.

The CARE guidelines were followed in the presentation of this case.

## Discussion and conclusions

There have been eight reported cases of SLC35A2-CDG with clinical details, including 2 males and 6 females, all caused by de novo mutations of *SLC35A2* [4, 6–8]. There is substantial variability in clinical symptoms within the group (Table 1). This variability could be due to the type of mutation (frameshift vs. missense), the level of mosaicism, or the degree of X-chromosome inactivation. Three female patients (our patient, patient 6 and patient 7) were found to have skewed X-inactivation. Kodera et al. found only the wild-type *SLC35A2* allele to be expressed in the lymphoblastoid cell lines of patients 6 and 7 predicting that further mutant alleles were silenced by the skewed X-chromosome inactivation [6]. Additionally, Dorre et al. discovered only wild-type alleles in fibroblasts and only mutated alleles in lymphocytes of patient 5 [7]. These findings suggest that complicated patterns of X-chromosome inactivation may play a role in phenotypic severity in SLC35A2-CDG. Additionally, both previously reported male patients (Table 1) were mosaic for a *SLC35A2* mutation, suggesting that this disorder may be X-linked dominant and that males must be mosaic for survival.

All reported patients, including the current one, had brain MRI abnormalities (Table 1) and had neurological symptoms ranging from developmental delay and hypotonia to early onset infantile encephalopathy with severe seizures and hypsarrhythmia. Only patient 1 (our patient) and patient 4 [4] who were both mosaic for the c.991G > A mutation in *SLC35A2*, did not have seizures (Table 1). Microcephaly was present in patients 3 and 4 [4] but not in our patient. Additionally, our patient does not have impaired kidney function, shortened limbs or severe gastrointestinal issues that were present in patient 4 [4].

Our patient demonstrated considerably more advanced neurodevelopmental function than previously described patients, many of whom were nonverbal and unable to walk [6, 7]. The invariable presence of central nervous system (CNS) deficits has led other authors to hypothesize that the negative selection of the mutant *SLC35A2* allele seen in most tissues may happen to a lesser degree in the CNS with most of the neurons expressing the mutant *SLC35A2* allele [6].

Transferrin isoform testing by mass spectrometry or isoelectric focusing to detect elevated levels of hypoglycosylated serum transferrins is the current practice for diagnosis of N-glycosylation defects [4, 5]. Our patient and four of the 8 other SLC35A2-CDG cases (Table 2) had abnormal transferrin glycosylation profiles before the first year of life, but testing had normalized by ages 1–3 years. A current hypothesis is that during infancy the body selects hepatocytes with the mutant allele [4]. Even in the most common CDG, PMM2-CDG, there have been two cases of normal or nearly normal transferrin levels in children: one child with only slightly abnormal and a second with abnormal transferrin levels at 1 year that normalized by age 8 [13]. Such normalization of transferrin IEF after the first year of life in some children with SLC35A2-CDG emphasizes the importance of performing WES in children with clinical findings compatible with CDG [14] but normal transferrin levels.

One predominant issue in patients with CDG is failure to thrive, which is thought to be caused by a combination of poor nutritional status and/or impairment of the GH-IGF-1 cascade due to hypoglycosylation of growth factors and their receptors. Significantly decreased levels of ALS, IGFBP3, IGF-1 and IGF-2, and ternary complex formation have been described in other patients with PMM2-CDG [15–17].

Dhaunsi [18] found low IGF-1 levels in 3 patients with CDG-II (specific mutations not specified) and selective impairment of IGF-1-induced synthesis of DNA in the lymphoblasts of children with either PMM2-CDG or CDG-II compared to controls. Of interest, both children with PMM2-CDG and with CDG-II had remarkable hypoglycosylation of their IGF-1 receptor protein compared to controls, which may explain the decreased IGF-1-induced synthesis of DNA, even in children with normal IGF-1 levels. The IGF-1 receptor is a glycoprotein and requires proper post-translational glycosylation of its protein for proper function [19]. Impaired IGF-1 receptor glycosylation and signaling may explain the growth failure seen in our patient in the presence of normal IGF-1 level and GH production. GH and/or IGF-1 therapies in children with CDG may have a role in improving the growth in children with CDG and growth failure.

This report provides additional information on the phenotypic spectrum of SLC35A2-CDG*,* a rare condition now described in 9 patients. Because of the rarity of CDG, regardless of type, data on linear growth in children with CDG is limited. This is the first report to evaluate the growth axis of CDG-IIm including a provocative GH stimulation test. Although our patient’s peak GH response was high and her growth hormone dependent IGF-1, IGFBP3, and ALS levels were within normal ranges, her growth and weight gain have continued to be below the 3rd percentile.

In summary, WES testing should be utilized to identify rare CDG disorders in children with normal transferrin levels, but who have other clinical findings that are usually seen in CDG.

## Additional files

Additional file 1: **Figure S1.** Sequencing data for *SLC35A2* mutation. [A]- Integrative Genomics Viewer image of next generation sequencing data showing chrX:g.48762195C > T (HG19) variant present in 20% of reads. [B] Bidirectional Sanger sequence confirmation of c.991G > A *SLC35A2* variant in peripheral blood sample from proband. [C] Bidirectional Sanger sequence data from maternal blood sample demonstrating absence of the c.991G > A variant. Note- next generation sequence data shown in relation to the HG19 chromosome X reference sequence, while Sanger sequencing data is presented in relation to the *SLC35A2* reference transcript (NM_005660.2) which is located on the opposite strand. (TIFF 1521 kb)

Additional file 2: **Figure S2.** A, B. A. Length growth over time. B. Weight growth over time. (ZIP 65 kb)

## Abbreviations

ALS: Acid labile subunit
AR: Androgen receptor
ASH: Asymmetric septal hypertrophy
CDG: Congenital disorder of glycosylation
ECG: Electrocardiogram
EEG: Electroencephalogram
GERD: Gastroesophageal reflux disease
GH: Growth hormone
IGF: Insulin like growth factor
IGFBP: insulin like growth factor binding protein
MRI: Magnetic resonance imaging
ND: Not described
PCR: Polymerase chain reaction
TSH: Thyroid stimulating hormone
VSD: Ventricular septal defect
WES: Whole exome sequencing
