# Supplementary figures and images for: Mosaicism of the UDP-Galactose transporter SLC35A2 in a female causing a congenital disorder of glycosylation: a case report
Source: BMC Med Genet. 2018 Jun 15;19:100. doi: 10.1186/s12881-018-0617-6 (PMC6003163; doi:10.1186/s12881-018-0617-6)

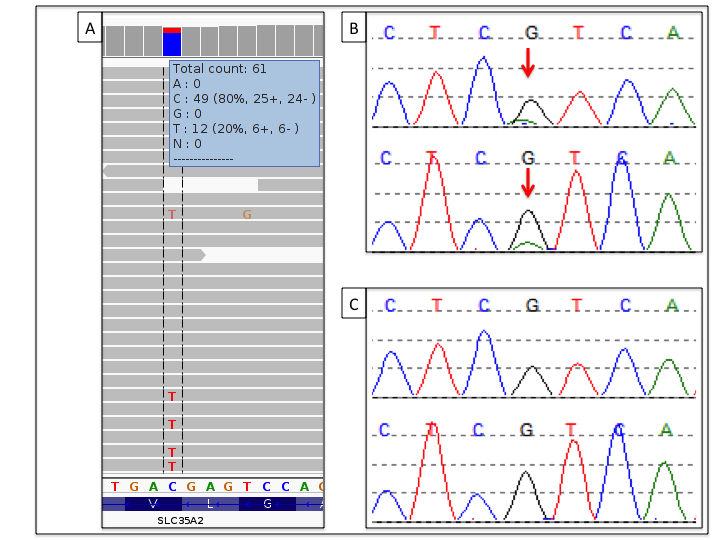

Supplement: Supplementary file 1 — Figure S1. Sequencing data for SLC35A2 mutation. [A]- Integrative Genomics Viewer image of next generation sequencing data showing chrX:g.48762195C > T (HG19) variant present in 20% of reads. [B] Bidirectional Sanger sequence confirmation of c.991G > A SLC35A2 variant in peripheral blood sample from proband. [C] Bidirectional Sanger sequence data from maternal blood sample demonstrating absence of the c.991G > A variant. Note- next generation sequence data shown in relation to the HG19 chromosome X reference sequence, while Sanger sequencing data is presented in relation to the SLC35A2 reference transcript (NM_005660.2) which is located on the opposite strand. (TIFF 1521 kb) [file 12881_2018_617_MOESM1_ESM.tiff]

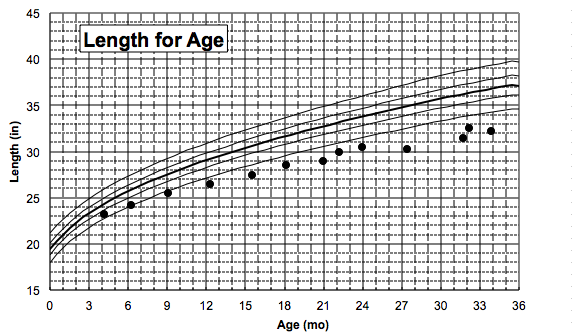

Supplement: Supplementary file 2 — Figure S2. A, B. A. Length growth over time. B. Weight growth over time. (ZIP 65 kb) [file 12881_2018_617_MOESM2_ESM.zip › Supplemental Fig.2a Westenfield et alR2.tiff]

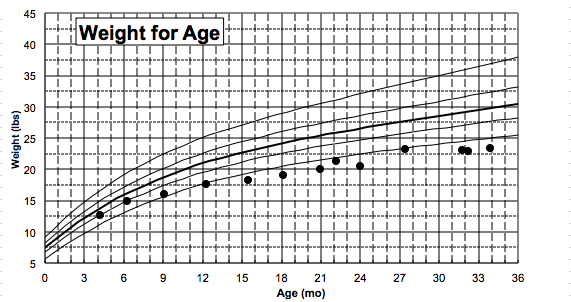

Supplement: Supplementary file 2 — Figure S2. A, B. A. Length growth over time. B. Weight growth over time. (ZIP 65 kb) [file 12881_2018_617_MOESM2_ESM.zip › Supplemental Fig.2b Westenfield et alR2.tiff]
